# A Systematic Review of the Burden of Pancreatic Cancer in Europe: Real-World Impact on Survival, Quality of Life and Costs

**DOI:** 10.1007/s12029-015-9724-1

**Published:** 2015-05-14

**Authors:** A. Carrato, A. Falcone, M. Ducreux, J. W. Valle, A. Parnaby, K. Djazouli, K. Alnwick-Allu, A. Hutchings, C. Palaska, I. Parthenaki

**Affiliations:** Medical Oncology Department, Ramon y Cajal University Hospital, Ctra. Colmenar Viejo Km. 9,100, Madrid, Spain; Unit of Medical Oncology, Pisa University Hospital, Via Roma 67, Pisa, 56126 Italy; Gastrointestinal Unit, Gustave Roussy Institute, 114 Rue Edouard-Vaillant, 94805 Villejuif, France; Department of Medical Oncology, University of Manchester and Christie NHS Foundation Trust, Wilmslow Rd, Manchester, M20 4BX UK; Celgene Corporation, Route de Perreux 1, 2017 Boudry, Switzerland; Dolon Ltd, 175-185 Grays Inn Road, London, WC1X 8UE UK

**Keywords:** Pancreatic cancer, Disease burden, Epidemiology, Survival, Quality of life, Proportional shortfall

## Abstract

**Purpose:**

The purpose of this study was to assess the overall burden of pancreatic cancer in Europe, with a focus on survival time in a real-world setting, and the overall healthy life lost to the disease.

**Methods:**

Real-world data were retrieved from peer-reviewed, observational studies identified by an electronic search. We performed two de novo analyses: a proportional shortfall analysis to quantify the proportion of healthy life lost to pancreatic cancer and an estimation of the aggregate life-years lost annually in Europe.

**Results:**

Ninety-one studies were included. The median, age-standardised incidence of pancreatic cancer per 100,000 was 7.6 in men and 4.9 in women. Overall median survival from diagnosis was 4.6 months; median survival was 2.8–5.7 months in patients with metastatic disease. The proportional shortfall analysis showed that pancreatic cancer results in a 98 % loss of healthy life, with a life expectancy at diagnosis of 4.6 months compared to 15.1 years for an age-matched healthy population. Annually, 610,000–915,000 quality-adjusted life-years (QALYs) are lost to pancreatic cancer in Europe. Patients had significantly lower scores on validated health-related quality of life instruments versus population norms.

**Conclusions:**

To the best of our knowledge, this is the first study to systematically review real-world overall survival and patient outcomes of pancreatic cancer patients in Europe outside the context of clinical trials. Our findings confirm the poor prognosis and short survival reported by national studies. Pancreatic cancer is a substantial burden in Europe, with nearly a million aggregate life-years lost annually and almost complete loss of healthy life in affected individuals.

**Electronic supplementary material:**

The online version of this article (doi:10.1007/s12029-015-9724-1) contains supplementary material, which is available to authorized users.

## Introduction

Pancreatic cancer is among the most deadly malignancies; despite being responsible for only 3 % of all new cancer diagnoses, it was the fourth most common cause of cancer death in the United States in 2013 [1] and is expected to be the second most common cause by 2030 [2]. The reported incidence is highest in developed countries, which likely reflect more accurate diagnosis, with Europe bearing a significant part of the burden [3]. In 2008, Europe carried one quarter of the global burden, despite comprising only one ninth of the world population [4]. Accurate data on cancer incidence and mortality in Europe are therefore crucial in assessing the burden of disease, the effectiveness of control programmes and for budgeting and planning at national and regional levels.

Current treatment options for pancreatic cancer are limited, with surgical resection presently the only potentially curative treatment option [5, 6]. However, owing to the frequently advanced stage at diagnosis, 80–90 % of patients have unresectable tumours [5], and long-term survival after surgical resection is poor [6]. New treatments have recently been approved and are expected: chemotherapy has demonstrated a favourable impact in overall survival when prescribed after surgery with curative intent [7]; gemcitabine and erlotinib are approved in the USA and Europe for use in metastatic disease; and *nab*-paclitaxel has recently been approved in Europe and the USA to treat metastatic disease. In order to improve long-term outcomes for pancreatic cancer patients, it is imperative that patients have access to the range of new treatment options. Key stakeholders in this process are healthcare payers. In order to support the introduction of these treatments, real-world survival times provide a meaningful context in which to evaluate improvements in patient outcomes. Survival times are an important way of capturing the unmet medical needs of a patient as they allow the calculation of shortfall. They also allow us to capture the unmet medical needs at a societal level through the number of aggregate life-years lost to the disease [8].

To our knowledge, no published studies have systematically reviewed the real-world survival time in Europe outside context of clinical trials. The objective of the systematic review was therefore to perform a systematic, macroscopic analysis of the burden of pancreatic cancer in Europe on individuals and health systems, to evaluate the direct and indirect costs of the disease, and to assess the extent of the unmet needs.

## Methods

The research comprised two phases: a systematic review of published data and related de novo analyses.

### Systematic Review Methods

Studies were identified through a search of MEDLINE and other major bibliographic databases for studies published in English through 5th April 2013. Additional information was obtained from searches of conference proceedings for the 2 years preceding April 2013 and targeted searches of health economic databases. Only epidemiological or non-interventional studies were selected for data extraction; eligibility and exclusion criteria are shown in Table 1 (see online resource for details of databases and search terms). Grey literature, such as unpublished reports from disease registry websites, was not included. Studies based on the same dataset were systematically excluded in order to avoid multiple reporting of the same data.

**Table 1:** Study inclusion and exclusion criteria

Study types eligible for inclusion	Study types that were excluded
• Observational/uncontrolled cohort studies (prospective/retrospective longitudinal studies, cross-sectional studies) • Studies with both observational and interventional phases • Database studies, registries • Studies with no available abstract	• Interventional trials (parallel and crossover design, double-blind, single-blind, open label) with no observational phases • Phase I, II, III or IV studies • Case reports or case series • Case–control studies • In vitro studies/genetic studies/molecular epidemiology studies • Imaging/diagnostic studies; studies assessing techniques • Classification schemes (e.g. studies assessing staging schemes) • Studies focusing on pathophysiology or prevention • Systematic reviews • Editorials, letters, comments, non-systematic review articles • Guidelines • Policy/prioritisation papers/research recommendations

Two independent reviewers performed the review and full-text extraction; any differences were resolved by a third reviewer. No quantitative syntheses, e.g. meta-analysis, were performed. Overall medians of median values reported in individual studies were calculated as summary measures.

### De Novo Analysis: Proportional Shortfall

Proportional shortfall (PS) was calculated as follows [9]:

$$ \mathrm{P}\mathrm{S}=\frac{\mathrm{disease}\kern0.5em \mathrm{related}\kern0.5em \mathrm{QALY}\kern0.5em \mathrm{loss}}{\mathrm{remaining}\kern0.5em \mathrm{QALY}\kern0.5em \mathrm{expectation}\kern0.5em \mathrm{in}\kern0.5em \mathrm{absence}\kern0.5em \mathrm{of}\kern0.5em \mathrm{the}\kern0.5em \mathrm{disease}} $$

Quality-adjusted life-year (QALY) expectation or ‘health life’ in the absence of pancreatic cancer was calculated by multiplying the mean life expectancy of patients aged 71 in EU 28 countries (15.1 years) [10], by the mean health-related quality of life (HRQoL) of this age group (0.78 = weighted health state index for the UK population aged 65–74) [11]. Pancreatic cancer-related QALYs were calculated by multiplying the median patient survival in months, derived from the current systematic review, by the EQ-5D utility obtained from a German study of 45 pancreatic cancer patients (mean EQ-5D utility 0.65; mean age 64 years) [12].

### De Novo Analysis: Aggregate Life-Years Lost

Aggregate life-years lost to pancreatic cancer was calculated from the difference in survival between pancreatic cancer patients and the general population multiplied by the annual number of incident pancreatic cancer cases across Europe obtained from three different sources: GLOBOCAN incident cases derived from crude incidence estimates (79,331 cases) [13]; crude incidence rates from the current systematic review multiplied by the EU 28 population of 507.2 million [14] (62,203 cases); and age-adjusted (European standard) rates derived from the current systematic review multiplied by the EU 28 population (52,872 cases). QALYs lost per person (calculated as per the PS analysis) were multiplied by the incident cases of pancreatic cancer in Europe to derive the aggregate QALYs lost. Analyses were performed for all patients with pancreatic cancer and metastatic patients (based on an estimated 62.5 % patients having Stage IV disease as a proportion of all patients in Stages I to IV [15]).

## Results

### Search and Selection of Studies

**Fig. 1:**
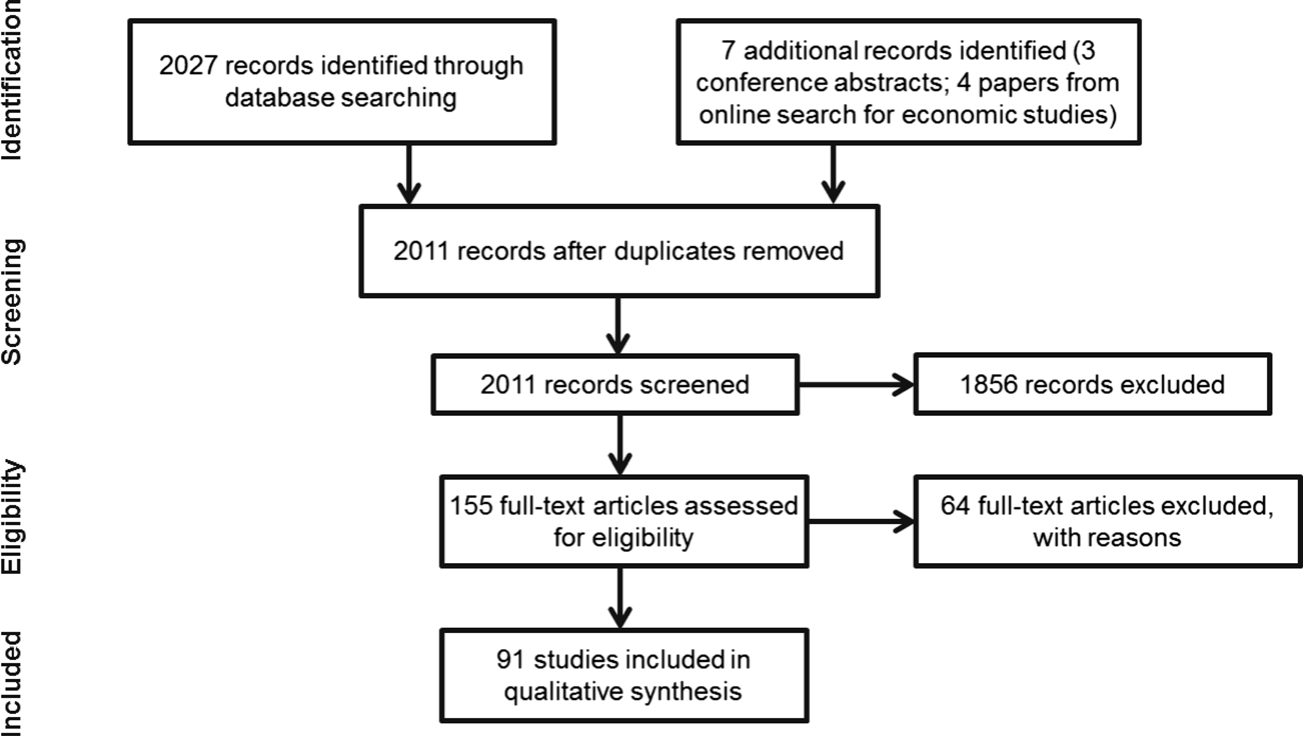
PRISMA flow diagram of the systematic review study selection process

The search of the electronic databases retrieved 2027 citations; 7 additional studies were identified in conference abstracts and economic databases (Fig. 1). After review, 91 studies met the inclusion criteria (study characteristics are described in online resources).

**Fig. 2:**
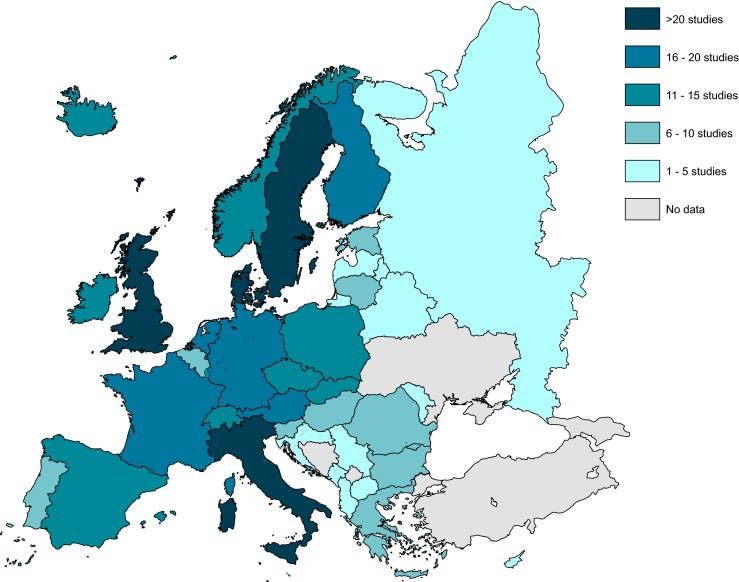
Geographic distribution of the selected epidemiological studies

Studies contained data from all regions of Europe, with some publications reporting data from several different counties and regions. The number of studies reporting data from the different countries is shown in Fig. 2. The Scandinavian countries, the UK and Italy were the most represented and the Baltic and Balkan states the least represented. Year of publication ranged from 1973 to 2013, with the majority (72 %) published in the last 10 years. Study durations ranged from <1 year to >60 years (see Table S1). In studies of patient registries, the number of patients with pancreatic cancer (where reported) ranged from 48 (regional registry) to 69,304 (WHO international registry) with 35 % containing >20,000 patients. Registries were not exclusively pancreatic cancer specific; individual registries also contained data for up to 1.76 million non-pancreatic cancer patients. In cohort, cross-sectional and other studies, the number of patients with pancreatic cancer ranged from 17 to 2196, with 88 % of studies including <500 patients. Where reported, the mean age of study subjects ranged from 50 to 75 years (median range 57 to 74 years). Six of the studies reported economic data and five reported data on HRQoL.

### Incidence of Pancreatic Cancer

Seventeen publications reported age-standardised incidence data from 37 study populations in 24 countries [16–32]. In the majority of populations (33/37), incidence rates were reported as age-standardised based on the World Standard Population (WSP) [16–28]; four studies reported age-standardised data based on the European Standard Population (ESP) [29–32], and five studies reported crude incidence rates [33–35, 28, 36]. Median annual incidence rates are shown in Table 2. Overall median annual incidence rates were higher in men than women for all age-adjustment standards.

**Table 2:** Median annual incidence rates of pancreatic cancer in Europe by sex: age-standardised and crude rates

	Annual incidence rate per 100,000: median (range)		
	Women	Men	Total^a^
Age-standardised: World (33 studies)	4.9 (3.2–9.8)	7.6 (5–14.1)	6.29
Age-standardised: Europe (4 studies)	9.2 (7.7–11.2)	11.8 (10–16.5)	10.43
Crude: (5 studies)	11.5 (6.1–14.7)	12.2 (8–15.4)	12.27

^a^Calculated with weighting based on 51 or 52 % male (as per study, or assumed if not reported)

### Median Survival

**Fig 3:**
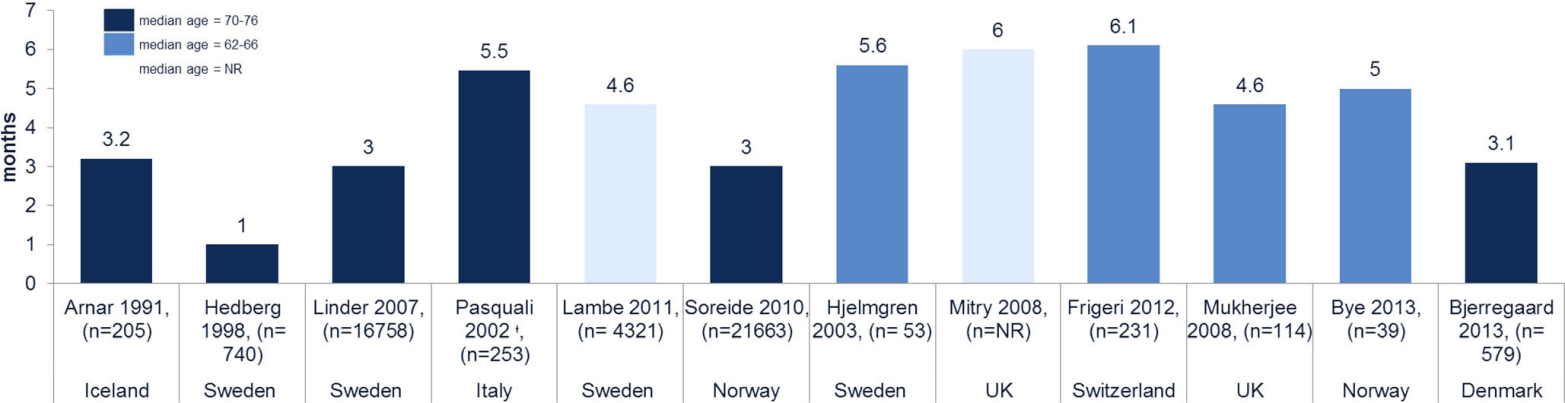
Median survival from diagnosis in months by median age. Data represents median survival of the overall study population ordered by midpoint of study period

Median survival from diagnosis was reported in 12 studies [26, 37, 38, 33, 34, 18, 39–44], mainly from Nordic countries (8/12 studies), and ranged from 1 to 6.1 months, with an overall median of 4.6 (Fig. 3). The two largest studies (in Norwegian and Swedish populations of 21,663 and 16,758 patients, respectively) both reported a median survival of 3 months [33, 18]. When reported, stage at diagnosis was generally advanced, with half the studies reporting >40 % of patients with metastatic disease [26, 34, 39–41, 43]. In general, median survival was shorter in older populations (1 to 3.2 months; median age 70–76 years) versus younger ones (5 to 6.1 months; median ages 62–67 years).

### Survival Rates at 1 and 5 Years

Combined survival data for both sexes were available from 32 studies or sub-studies reported in 17 publications [45–49, 44, 34, 50, 37, 38, 33, 51–54, 29, 43] (Figure S1 in the online resource). Combined 1-year survival rates ranged from 10 to 23 %. The largest study in a population of 30,025 Dutch patients [49] reported a 1-year survival rate of 16 %. The overall median 1-year survival across studies was 15 %.

Combined 5-year survival rates ranged from 0.5 % in Sweden [37] to 9 % in Slovenian [46] and German populations [47]. The two largest studies reported 5-year survival rates of 3 and 5 % [49, 48]. Overall median 5-year survival across studies was 3 %.

### Survival Rates at 1 and 5 Years by Sex

Survival rates at 1 and 5 years by sex were reported in 33 studies or sub-studies in nine publications [53, 36, 38, 42, 55, 18, 56, 31, 48]. Overall median values across studies were 15 % in women and 14 % in men at 1-year and 4 % in both sexes at 5 years (Table 3).

**Table 3:** Survival at 1 and 5 years from diagnosis

33 studies	Survival (%): median (range)	
	Women	Men
1 year	15 (9 [Denmark]–29 [Malta])	14 (9 [Malta]–22 [Sweden and Finland])
5 years	4 (1.3 [Slovenia]–7.5 [Czech Rep.)	4 (0 [Switzerland]–7 [Estonia])

### Survival by Disease Stage

The effect of disease stage (irrespective of intervention) on median survival from diagnosis was reported by three studies [41, 43, 39]. As expected, survival rates decreased with advancing stage. In a Dutch population between 1985 and 2001, survival rates at 1 year were 40, 30, 35, 25 and 5 % for stages I, II, III, IVa and IVb, respectively [15]. In a Finnish population between 1947 and 1980, 1-year survival rates were 53, 19, 17 and 12 % for stages I, II, III and IV, respectively [53]. In the aforementioned Dutch population, survival at 5 years was 15, 1, 5 and 1 % among patients with stages I, II, III and IV disease, respectively [15].

### Median Survival by Intervention

Five studies reported survival from diagnosis by intervention; four were in Scandinavian populations [44, 37, 38, 33] and one in an Italian population [34]. Patients who underwent resection or radical surgery consistently showed the longest median survival, ranging from 11 to 25.7 months. In patients who underwent chemotherapy, radiotherapy, palliative surgery or exploratory laparotomy, median survival ranged from 2 to 8.1 months. Patients who received no surgery or best supportive care had a median survival of 1.1 month (Figure S2 in the online resource).

The shorter survival among patients treated with chemotherapy, radiotherapy, palliative surgery or exploratory laparotomy reflects the more advanced stage of disease in these patients (54 % of patients treated with chemotherapy/radiotherapy and 52 % with best supportive care had stage IV disease, versus 3 % of patients receiving resective/radical surgery).

### Mortality

Sixteen studies reported age-standardised mortality rates, based on the WSP [57–66, 17, 21, 67] or ESP [68, 17, 31], and five studies reported crude rates [52, 63, 69, 64, 21]. Mortality data were derived almost solely from the World Health Organisation (WHO) and EUROCARE registries. In pan-European registry studies, median rates were from 5.0 per 100,000 in women and 7.7 per 100,000 in men, with similar rates across the EU5, Nordics and Eastern European regions. The highest median rates in men and women were recorded in Eastern Europe and the Nordic regions, respectively (Table 4).

**Table 4:** Annual mortality rate per 100,000 in Europe as whole and in European regions

Age-standardised, world (124 studies/sub-studies)	Annual mortality rate per 100,000: median (range)	
	Women	Men
Pan-European	5.0 (4.7–5.4)	7.7 (7.2–8)
EU5	4.9 (2.9–5.8)	7.4 (5.3–8.5)
Nordic	5.4 (5.2–7.9)	8.2 (6.8–9.9)
Eastern European	4.9 (2.8–7.2)	8.7 (6.3–12.3)

Age-standardised mortality rates based on the ESP in women and men ranged from 7.4 to 9.7 per 100,000, respectively, in England/Wales to 11.8 and 15.3, respectively, in the Netherlands [68, 30, 31]. Crude mortality rates per 100,000 ranged from 11.2 [52] to 16.7 [63] in women and from 12.5 [69] to 16.5 [63, 64] in men.

Data were not evenly distributed over time, with the majority coming from the period 2000 to 2006. Women had lower mortality rates than men at all time periods where data were collected.

### HRQoL

**Fig. 4:**
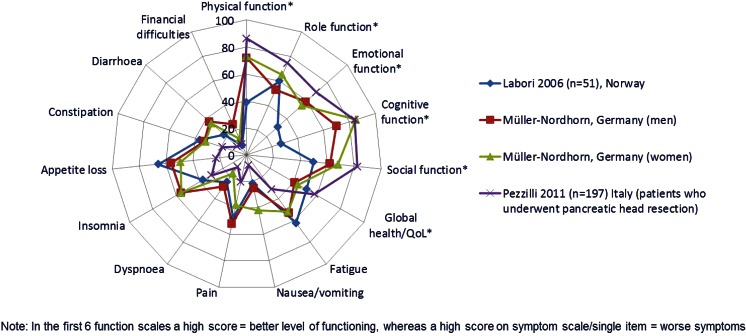
Results of the EORTC QLQ-30 questionnaire from four studies

Five studies reported HRQoL data [40, 70, 12, 71, 72]. In general, patients with pancreatic cancer had significantly lower scores on validated European Organisation for Research and Treatment of Cancer (EORTC) HRQoL scales than the general population, with the most significant symptoms being pain, appetite loss and insomnia. Patients with pancreatic cancer experienced depression and anxiety; cognitive, social and physical function tended to be high, while global health was low (Fig. 4).

Three studies evaluated depression and anxiety in patients with advanced disease [70, 40, 12]. In a Swedish population, 42 % of patients reported moderate or severe anxiety and depression. In a German study, the number of patients experiencing anxiety/depression was around 10-fold higher than population norms [12].

### Economic Burden

Three studies reported direct costs [39, 73, 74] and two indirect costs [73, 74] associated with pancreatic cancer. In Sweden, the mean monthly total cost of care from 2005 to 2007 was just over €6500 per month [74], corresponding to €16,066 over the residual lifetime. In Germany, total costs were considerably higher at €31,375 over the residual lifetime or €48,900 per year [73]. Data on the relative contribution of indirect versus direct costs to the overall cost were not consistent between countries. In Sweden, indirect costs accounted for a greater proportion of overall costs than direct costs (€83,109 versus 16,066, respectively) [74], whereas the opposite was reported in Germany (€28,164 versus 3210) [73].

Five studies reported direct cost data [75, 39, 73, 34, 74]. Hospitalisation accounted for the major component of direct costs per residual lifetime (€7981–16,264), followed by interventions (radiology, surgery and chemotherapy; €1575–9761) and chemotherapy alone (€1423–3569).

Two studies reported indirect costs [73, 74]. In Germany, 24 % of diagnosed patients were actively employed, resulting in a mean productivity loss of €2972 [73]. In Sweden in 2009, indirect costs were slightly higher for male versus female patients aged ≤64 years, with mean short-term productivity loss of €87,205 for men and €49,895 for women; mean productivity loss per patient due to mortality was €238,843 in men and €220,543 in women [74].

### De Novo Analysis of Proportional Shortfall

Mean age for diagnosis of pancreatic cancer was 71 years. In Europe, the general population aged 71 can expect an additional 11.78 years of healthy life, compared with only 0.25 years for patients with pancreatic cancer (0.38 years of life expectancy multiplied by 0.65 [12]). This corresponds to an absolute shortfall of 11.53 QALYs, and a proportional shortfall of 0.98 (11.53/11.78), indicating a 98 % loss of expected healthy life (Table 5).

**Table 5:** Proportional shortfall due to pancreatic cancer

	General population	Pancreatic cancer patients	Difference
Life expectancy at age 71	15.1 years	0.38 years (4.6 months)	14.7 years
Utility (ages 65–74)	0.78	0.65	0.13
QALYs	11.78 (A)	0.25 (B)	11.53 (C)
Proportional shortfall (C)/(A)			0.98

### De Novo Analysis of Aggregate Life-Years Lost

At 71 years of age, the survival difference (not adjusted by QoL) between a patient with pancreatic cancer and the general population is 14.7 years of life per patient (Table 5). Using GLOBOCAN data on the number of incident pancreatic cancer cases [76], this equates to over 1 million life-years lost across Europe (Table 6) or at least 778,000 life-years lost using the incidence estimates obtained in the present review.

**Table 6:** Aggregate life-years and QALYs lost to pancreatic cancer across Europe (EU 28)

	Incident cases^a^	Aggregate life-years lost	Aggregate QALYs lost
All pancreatic cancer			
Globocan (crude)	79,331	1,167,488	914,594
Systematic review (crude)	62,203	915,428	717,134
Systematic review (ESP)	52,872	778,095	609,549
Metastatic pancreatic cancer			
Globocan (crude)	44,901	663,792	519,606
Systematic review (crude)	35,207	520,479	407,423
Systematic review (ESP)	29,925	442,397	346,301

*ESP* age adjusted with European Standard Population

^a^Metastatic incident cases based on 62.5 % of patients in Stages IV, a figure scaled up from that in the original publication in order that proportions of patients in the study in Stages I to IV sum to 100 % [15]

## Discussion

The clinical studies identified represented all regions of Europe, although the North of Europe, in particular Scandinavian countries, represented almost half studies. Survival times in pancreatic cancer are very short; this is partly explained by the lack of effective treatments and the difficulties in diagnosing the disease early. Data from large cohort studies and national/regional registries showed 1- and 5-year survival rates of about 15 and 4 %, respectively. Our estimated median survival from diagnosis of 4.6 months is in line with a median of 3 months reported in a US study of 32,452 adults with distant metastatic pancreatic cancer [77] and 10 weeks in a recent Dutch study of 3099 patients with metastatic disease [78] and reflects the high number of patients with metastatic disease at diagnosis. The longest median survival from diagnosis was reported in patients who underwent resection or radical surgery. It is likely, however, that these data are skewed by disease stage, as patients who underwent resection are more likely to have had earlier stage disease.

We found that age-standardisation methodology had a notable influence on the incidence rate, with median rates ranging from approximately 8–16 per 100,000 annually in men, and 5–15 per 100,000 in women, depending on the method used. Incidence rate standardised to the WSP gave the lowest combined incidence (6.29/100,000), which was similar to those reported by GLOBOCAN (7/100,000) [76], which includes estimates from all EU countries. The combined crude incidence rate estimated from five studies was higher at 12.27/100,000 annually. Crude incidence rates give a picture of the actual rate in a population. However, incidence (and other rates) of cancer are strongly age-dependent; therefore, comparisons of crude rates between populations may be misleading if the age composition of the populations differ. Although age-standardisation facilitates comparisons, standardised rates can be deceiving if the age structure of the reference population is different to that of the real population. Owing to the substantially lower rates observed with age-adjustment to the WSP, we used only crude estimates or figures adjusted to ESP in the de novo analyses.

Mortality rates were higher in men than in women in all the studies identified; given the similar survival rates between the sexes, this is most likely driven by the higher incidence in men. The majority of studies reported mortality rates as age-standardised based on the WSP; however, similar to the incidence data, this resulted in substantially lower rates than ESP age-adjusted and crude estimates, making it questionable whether this standardisation method is appropriate for the European population. Mortality rates across European regions and individual countries were comparable to the Europe-wide figures. However, one study that used data from the WHO database reported appreciably higher mortality rates in eastern European accession countries compared with the European Union [79].

Expectedly, HRQoL scores were significantly lower and levels of depression and anxiety higher in patients with pancreatic cancer compared with population norms, indicating that there is scope to improve supportive care of patients until screening or early diagnosis is improved, or treatments that provide clear survival advantages become available.

A limitation of the current study is the lack of quantitative analysis, such as meta-analysis, of the epidemiological data. Nonetheless, we were able to quantify the aggregate number of life-years and QALYs lost due to pancreatic cancer in Europe each year. Various methods of incorporating equity concerns into standard QALY-based economic evaluations have been implemented, such as considering different incremental cost-effectiveness ratio (ICER) thresholds [80]. The PS approach that we have used includes an equity-weighting component that demonstrates the nearly complete loss of healthy life associated with pancreatic cancer. Recent developments such as value-based pricing in the UK and policies in other countries reflect an increasing willingness of decision-makers to account for such weightings in QALY-related assessments [81, 82]. The estimates of aggregate life-years and QALYs lost to pancreatic cancer demonstrate the magnitude of the disease’s impact in Europe and raise the question of how health systems are managing this burden.

We found few studies that assessed the cost of pancreatic cancer; in those identified, methodologies varied between studies. Hospitalisation accounted for the major component of direct costs, followed by interventions and chemotherapy, with some variation between countries. This was consistent with data from a large US study showing that hospitalisations and cancer-directed procedures accounted for the largest fraction of health care costs [83].

## Conclusions

The findings of this systematic review show that the real-world median survival time of patients with pancreatic cancer in Europe was less than 5 months and less than 10 % of patients survived beyond 5 years. Survival at 1 year was highest in patients with early stage disease who undergo resection, highlighting the need for improved early diagnosis. Our results confirm those of individual pan-European registry studies and US studies that report similar incidence and mortality rates for pancreatic cancer, reflecting the poor prognosis. Our analyses demonstrate the considerable burden of pancreatic cancer in Europe, with almost a million aggregate life-years lost and an almost complete loss of healthy life in affected individuals. Quantifying the number of life-years and QALYs lost provides a method to compare the relative burden of different cancers in the context of value-based pricing and to evaluate treatment options in view of the projected increase in disease burden [2] over the next 15 years.

## Electronic Supplementary Material

ESM 1 (DOCX 399 kb)
