# Supplementary material for: A Systematic Review of the Burden of Pancreatic Cancer in Europe: Real-World Impact on Survival, Quality of Life and Costs
Source: J Gastrointest Cancer. 2015 May 14;46(3):201–11. doi: 10.1007/s12029-015-9724-1 (PMC4519613; doi:10.1007/s12029-015-9724-1)
Supplement: Supplementary file 1 — (DOCX 399 kb) [file 12029_2015_9724_MOESM1_ESM.docx]

**A systematic review of the burden of pancreatic cancer in Europe: real-world impact on survival, quality-of-life, and costs**

A. Carrato, A. Falcone, M. Ducreux, J. W. Valle, A. Parnaby, K. Djazouli, K. Alnwick-Allu, A. Hutchings, C. Palaska, I. Parthenaki

**Journal of Gastrointestinal Cancer**

Corresponding author: Ms. Ioanna Parthenaki, Dolon Ltd, 175–185 Grays Inn Road, London WC1X 8UE, United Kingdom. +44 (0) 7545 875 817; ioanna.parthenaki@dolon.com

Supplementary data

**Databases and search terms**

We searched MEDLINE (including MEDLINE In-Process, Other Non-Indexed Citations), Embase, and Scopus for studies in English up to 5th April 2013 and conference proceedings from ASCO/ASCO GI and ESMO/World GI for the two years preceding April 2013.

The following search terms were used, structured according to each database (including MESH terms as applicable): pancreatic cancer AND (burden OR epidemiology OR morbidity OR prognosis OR quality-of-life OR utility OR cost OR cost-benefit analysis OR survival OR mortality OR statistics OR incidence OR prevalence OR predict OR forecast) AND (observational OR cohort OR uncontrolled OR prospective OR retrospective OR longitudinal OR cross sectional OR survey OR database OR registry OR economic).

To identify additional economic studies, we searched the UK NHS Economic Evaluation Database (EED), the Database of Abstracts of Reviews of Effects (DARE) database from the Centre for Reviews and Dissemination (CRD), and the United Kingdom’s National Health Service Evidence database (NHS Evidence) up to 5^th^ April 2013, using solely the phrase ‘pancreatic cancer’ and associated terms.

The International Society for Pharmacoeconomics and Outcomes Research (ISPOR) conference proceedings from the two years preceding April 2013 were also searched using the following terms: pancreatic cancer AND (economic OR cost OR productivity OR resource OR budget OR model OR “health technology assessment”).

**Studies selected for data extraction**

Aareleid T, Rahu M: Cancer survival in Estonia from 1978 to 1987. Cancer 1991, 68:2088-2092.

Adanja B, Vlajinac H, Jarebinski M, Jovanovic D, Sipetic S, Marinkovic J: Trends in cancer mortality of the digestive tract in Belgrade, Yugoslavia, 1975-1989. Eur J Epidemiol 1994, 10:99-104.

Allen-Mersh TG, Earlam RJ: Pancreatic cancer in England and Wales: surgeons look at epidemiology. Ann R Coll Surg Engl 1986, 68:154-158.

Appelqvist P, Viren M, Minkkinen J, Kajanti M, Kostiainen S, Rissanen P: Operative finding, treatment, and prognosis of carcinoma of the pancreas: an analysis of 267 cases. J Surg Oncol 1983, 23:143-150.

Arfe A, Malvezzi M, Bertuccio P, Decarli A, La Vecchia, C, Negri E: Cancer mortality trend analysis in Italy, 1970-2007. Eur J Cancer Prev 2011, 20:364-374.

Arnar DO, Theodors A, Isaksson HJ, Gunnlaugsson GH, Tulinius H, Johannsson H, Kjartansson S: Cancer of the pancreas in Iceland. An epidemiologic and clinical study, 1974-85. Scand J Gastroenterol 1991, 26:724-730.

Baastrup R, Sorensen M, Hansen J, Hansen RD, Wurtzen H, Winther JF: Social inequality and incidence of and survival from cancers of the oesophagus, stomach and pancreas in a population-based study in Denmark, 1994-2003. Eur J Cancer 2008, 44:1962-1977.

Bachmann M, Peters T, Harvey I: Costs and concentration of cancer care: evidence for pancreatic, oesophageal and gastric cancers in National Health Service hospitals. J Health Serv Res Policy 2003, 8:75-82.

Bernal M, Gomez FJ, Gomez G: Trends in cancer mortality in Spain: 1975-2004. Tumori 2009, 95:669-674.

Bjerregaard JK, Mortensen MB, Schonnemann KR, Pfeiffer P: Characteristics, therapy and outcome in an unselected and prospectively registered cohort of pancreatic cancer patients. Eur J Cancer 2013, 49:98-105.

Borras J, Borras JM, Galceran J, Sanchez V, Moreno V, Gonzalez JR: Trends in smoking-related cancer incidence in Tarragona, Spain, 1980-96. Cancer Causes Control 2001, 12:903-908.

Bosetti C, Bertuccio P, Negri E, La Vecchia C, Zeegers MP, Boffetta P (2012) Pancreatic cancer: overview of descriptive epidemiology. Molecular carcinogenesis 2012, 51:3-13.

Bramhall SR, Allum WH, Jones AG, Allwood A, Cummins C, Neoptolemos JP: Treatment and survival in 13 560 patients with pancreatic cancer, and incidence of the disease, in the West Midlands: An epidemiological study. Brit J Surg 1995, 82:111-115.

Bray I, Brennan P, Boffetta P: Projections of alcohol- and tobacco-related cancer mortality in Central Europe. Int J Cancer 2000, 87:122-128.

Bye A, Jordhøy MS, Skjegstad G, Ledsaak O, Iversen PO, Hjermstad MJ: Symptoms in advanced pancreatic cancer are of importance for energy intake. Supportive Care in Cancer 2013, 21:219-227.

Carpelan-Holmstrom M, Nordling S, Pukkala E, Sankila R, Luttges J, Kloppel G, Haglund C: Does anyone survive pancreatic ductal adenocarcinoma? A nationwide study re-evaluating the data of the Finnish Cancer Registry. Gut 2005, 54:385-387.

Coupland VH, Kocher HM, Berry DP, Allum W, Linklater KM, Konfortion J, Moller H, Davies EA: Incidence and survival for hepatic, pancreatic and biliary cancers in England between 1998 and 2007. Cancer Epidemiol 2012, 36:e207-e214.

Crocetti E, Capocaccia R, Casella C, Guzzinati S, Ferretti S, Rosso S, Sacchettini C, Spitale A, Stracci F, Tumino R: Population-based incidence and mortality cancer trends (1986-1997) from the network of Italian cancer registries. Eur J Cancer Prev 2004, 13:287-295.

Cronin-Fenton DP, Erichsen R, Mortensen FV, Dikinis S, Norgaard M, Jacobsen J: Pancreatic cancer survival in central and northern Denmark from 1998 through 2009: A population based cohort study. Clin Epidemiol 2011, 3:19-25.

Cylwik B, Nowak HF, Glowinska L: Malignant neoplasms of the pancreas. A study based on autopsy data from 1953 to 1982 in Bialystok, Poland. I. Frequency, age and sex distribution. Neoplasma 1984, 31:597-603.

Decarli A, La Vecchia, C, Mezzanotte G, Cislaghi C: Birth cohort, time, and age effects in Italian cancer mortality. Cancer 1987, 59:1221-1232.

Dernedde U, Dernedde R, Shepstone L, Barrett A: Three-year single institution audit on transfusion requirements in oncology patients. Clin Oncol (R Coll Radiol) 2007, 19:223-227.

Desauw C, El Hajbi F, Ligier K, Duhamel A, Richard F, Rose C: Pathological proof and survival for patients with billiary-tract or pancreatic tumor. Presented at the annual meeting of the European Society for Medical Oncology (ESMO), 28 Sep - 02 Oct 2012, Vienna, Austria 2012.

Downing A, Forman D, Gilthorpe MS, Edwards KL, Manda SO: Joint disease mapping using six cancers in the Yorkshire region of England. Int J Health Geogr 2008, 7:41.

Faivre J, Forman D, Esteve J, Obradovic M, Sant M: Survival of patients with primary liver cancer, pancreatic cancer and biliary tract cancer in Europe. EUROCARE Working Group. Eur J Cancer 1998, 34:2184-2190.

Fernandez E, La Vecchia, C, Porta M, Negri E, Lucchini F, Levi F: Trends in pancreatic cancer mortality in Europe, 1955-1989. Int J Cancer 1994, 57:786-792.

Fitzsimmons D, Johnson CD, George S, Payne S, Sandberg AA, Bassi C, Beger HG, Birk D, Buchler MW, Dervenis C, et al: Development of a disease specific quality of life (QoL) questionnaire module to supplement the EORTC core cancer QoL questionnaire, the QLQ-C30 in patients with pancreatic cancer. EORTC Study Group on Quality of Life. Eur J Cancer 1999, 35:939-941.

Fitzsimmons D, Osmond C, George S, Johnson CD: Trends in stomach and pancreatic cancer incidence and mortality in england and wales 1951-2000, analysis by age, period and cohort. Pancreas 2006, 33:461.

Frigeri M, De Dosso S, Castillo-Fernandez O, Feuerlein K, Neuenschwander H, Saletti P: Chemotherapy in patients with advanced pancreatic cancer: Too close to death? Supportive Care in Cancer 2013, 21:157-163.

Gencer D, Kastle-Larralde N, Pilz LR, Weiss A, Buchheidt D, Hochhaus A, Hofheinz RD: Presentation, treatment, and analysis of prognostic factors of terminally ill patients with gastrointestinal tumors. Onkologie 2009, 32:380-386.

Grieve DC: Adenocarcinoma of the pancreas. A review of 100 cases. J R Coll Surg Edinb 1973, 18:221-226.

Hakama M, Hakulinen T, Teppo L, Saxen E: Incidence, mortality or prevalence as indicators of the cancer problem. Cancer 1975, 36:2227-2231.

Hariharan D, Saied A, Kocher HM: Analysis of mortality rates for pancreatic cancer across the world. HPB 2008, 10:58-62.

Haynes K, Forde KA, Schinnar R, Wong P, Strom BL, Lewis JD: Cancer incidence in The Health Improvement Network. Pharmacoepidemiol Drug Saf 2009, 18:730-736.

Hedberg M, Borgstrom A, Genell S, Janzon L: Survival following pancreatic carcinoma: a follow-up study of all cases recorded in Malmo, Sweden, 1977-1991. Brit J Surg 1998, 85:1641-1644.

Hill C, Benhamou E, Doyon F: Trends in cancer mortality, France 1950-1985. Br J Cancer 1991, 63:587-590.

Hippisley-Cox J, Coupland C: Identifying patients with suspected pancreatic cancer in primary care: derivation and validation of an algorithm. BrJ GenPract 2012, 62:e38-e45.

Hiripi E, Gondos A, Emrich K, Holleczek B, Katalinic A, Luttmann S, Sirri E, Brenner H: Survival from common and rare cancers in Germany in the early 21st century. Annals of oncology : official journal of the European Society for Medical Oncology / ESMO 2012, 23:472-479.

Hjelmgren J, Ceberg J, Persson U, Alvegard TA: The cost of treating pancreatic cancer--a cohort study based on patients' records from four hospitals in Sweden. Acta Oncol 2003, 42:218-226.

Jacobsen O, Olsen SW, Nielsen NA: Pancreatic cancer in the Faroe Islands. An epidemiologic study of patients with pancreatic cancer in the Faroe Islands 1972-82. Scand J Gastroenterol 1985, 20:1142-1146.

Jooste V, Remontet L, Colonna M, Belot A, Launoy G, Binder F, Faivre J, Bouvier AM: Trends in the incidence of digestive cancers in France between 1980 and 2005 and projections for the year 2010. Eur J Cancer Prev 2011, 20:375-380.

Karim-Kos HE, de Vries, E, Soerjomataram I, Lemmens V, Siesling S, Coebergh JW: Recent trends of cancer in Europe: a combined approach of incidence, survival and mortality for 17 cancer sites since the 1990s. Eur J Cancer 2008, 44:1345-1389.

Karim-Kos HE, Kiemeney LA, Louwman MW, Coebergh JW, de Vries, E: Progress against cancer in the Netherlands since the late 1980s: an epidemiological evaluation. Int J Cancer 2012, 130:2981-2989.

Kirkegaard J: Incidence of pancreatic cancer in Greenland 2000-2010. IntJ CircumpolarHealth 2012, 71:18368.

Klint A, Engholm G, Storm HH, Tryggvadottir L, Gislum M, Hakulinen T, Bray F: Trends in survival of patients diagnosed with cancer of the digestive organs in the Nordic countries 1964-2003 followed up to the end of 2006. Acta Oncol 2010, 49:578-607.

La Rosa F, Petrinelli AM, Minelli L, Mastrandrea V: Ten-year survival of patients with cancer of the digestive tract in Umbria, Italy. Eur J Epidemiol 1997, 13:9-13.

Labori KJ, Hjermstad MJ, Wester T, Buanes T, Loge JH: Symptom profiles and palliative care in advanced pancreatic cancer: a prospective study. Support Care Cancer 2006, 14:1126-1133.

Lambe M, Eloranta S, Wigertz A, Blomqvist P: Pancreatic cancer; reporting and long-term survival in Sweden. Acta Oncol 2011, 50:1220-1227.

Lepage C, Remontet L, Launoy G, Tretarre B, Grosclaude P, Colonna M, Velten M, Buemi A, Danzon A, Molinie F, et al: Trends in incidence of digestive cancers in France. Eur J Cancer Prev 2008, 17:13-17.

Levi F, La Vecchia, C, Decarli A, Randriamiharisoa A: Effects of age, birth cohort and period of death on Swiss cancer mortality, 1951-1984. Int J Cancer 1987, 40:439-449.

Levi F, La Vecchia, C, Randimbison L: Cancer mortality in Switzerland, 1990-1994. Soz Praventivmed 1997, 42:37-54.

Levi F, Lucchini F, Negri E, Boyle P, La Vecchia, C: Changed trends of cancer mortality in the elderly. Ann Oncol 2001, 12:1467-1477.

Levi F, Lucchini F, Negri E, Boyle P, La Vecchia, C: Mortality from major cancer sites in the European Union, 1955-1998. Ann Oncol 2003, 14:490-495.

Levi F, Lucchini F, Negri E, La Vecchia, C: Pancreatic cancer mortality in Europe: the leveling of an epidemic. Pancreas 2003, 27:139-142.

Levi F, Randimbison L, Te VC, Franceschi S, La Vecchia, C: Trends in survival for patients diagnosed with cancer in Vaud, Switzerland, between 1974 and 1993. Ann Oncol 2000, 11:957-963.

Linder S, Bostrom L, Nilsson B: Pancreatic carcinoma incidence and survival in Sweden in 1980-2000: a population-based study of 16,758 hospitalized patients with special reference to different therapies. Eur J Surg Oncol 2007, 33:616-622.

Liszka L, Pajak J, Mrowiec S, Zielinska-Pajak E, Lampe P, Golka D: Age distribution patterns of patients with conventional ductal adenocarcinoma of the pancreas. A single-institution study of 580 cases re-evaluated using current histopathological diagnostic criteria. Pol J Pathol 2010, 61:65-77.

Lombard-Bohas C, Mitry E, O'Toole D, Louvet C, Pillon D, Cadiot G, Borson-Chazot F, Aparicio T, Ducreux M, Lecomte T, et al: Thirteen-month registration of patients with gastroenteropancreatic endocrine tumours in France. Neuroendocrinology 2009, 89:217-222.

Luo J, Adami HO, Reilly M, Ekbom A, Nordenvall C, Ye W: Interpreting trends of pancreatic cancer incidence and mortality: a nation-wide study in Sweden (1960-2003). Cancer Causes Control 2008, 19:89-96.

Malvezzi M, Arfe A, Bertuccio P, Levi F, La Vecchia, C, Negri E: European cancer mortality predictions for the year 2011. Ann Oncol 2011, 22:947-956.

Malvezzi M, Bertuccio P, Levi F, La Vecchia, C, Negri E: European cancer mortality predictions for the year 2012. Ann Oncol 2012, 23:1044-1052.

Markovic-Denic L, Vlajinac H, Zivkovic S, Miljus D: Cancer mortality among men in Central Serbia: 1985-2006 survey study. Croat Med J 2008, 49:792-798.

Matos JM, Schmidt CM, Turrini O, Agaram NP, Niedergethmann M, Saeger HD, Merchant N, Johnson CS, Lillemoe KD, Grutzmann R: Pancreatic acinar cell carcinoma: a multi-institutional study. J Gastrointest Surg 2009, 13:1495-1502.

Micheli A, Ciampichini R, Oberaigner W, Ciccolallo L, de Vries, E, Izarzugaza I, Zambon P, Gatta G, De Angelis, R: The advantage of women in cancer survival: an analysis of EUROCARE-4 data. Eur J Cancer 2009, 45:1017-1027.

Mitry E, Rachet B, Quinn MJ, Cooper N, Coleman MP: Survival from cancer of the pancreas in England and Wales up to 2001. Br J Cancer 2008, 99:S21-S23.

Moulard O, Mehta J, Rose M, Olivares R, Hamed A, Eckert L: Survival estimates of six major advanced and metastatic solid tumors in europe: A cancer registry and literature review. Presented at the 15th Annual European Congress of the International Society for Pharmacoeconomics and Outcomes (ISPOR), November, 2012 in Berlin, Germany. 2012.

Mukherjee S, Hudson E, Reza S, Thomas M, Crosby T, Maughan T: Pancreatic cancer within a UK cancer network with special emphasis on locally advanced non-metastatic pancreatic cancer. Clin Oncol(R Coll Radiol) 2008, 20:535-540.

Müller-Nordhorn J, Bruggenjurgen B, Bohmig M, Selim D, Reich A, Noesselt L, Roll S, Wiedenmann B, Willich SN: Direct and indirect costs in a prospective cohort of patients with pancreatic cancer. Alim Pharmacol Ther 2005, 22:405-415.

Müller-Nordhorn J, Roll S, Bohmig M, Nocon M, Reich A, Braun C, Noesselt L, Wiedenmann B, Willich SN, Bruggenjurgen B: Health-related quality of life in patients with pancreatic cancer. Digestion 2006, 74:118-125.

Nagenthiraja K, Ewertz M, Engholm G, Storm HH: Incidence and mortality of pancreatic cancer in the Nordic countries 1971-2000. Acta Oncol 2007, 46:1064-1069.

Nienhuijs SW, van den Akker SA, de Vries, E, de Hingh IH, Visser O, Lemmens VE: Nationwide improvement of only short-term survival after resection for pancreatic cancer in the Netherlands. Pancreas 2012, 41:1063-1066.

Pasquali C, Sperti C, Filipponi C, Pedrazzoli S: Epidemiology of pancreatic cancer in Northeastern Italy: incidence, resectability rate, hospital stay, costs and survival (1990-1992). Dig Liver Dis 2002, 34:723-731.

Patterson CC, Kee F: Geographical variations and recent trends in cancer mortality in Northern Ireland (1979-88). Ulster Med J 1991, 60:137-149.

Pezzilli R, Falconi M, Zerbi A, Casadei R, Valli L, Varale R, Armatura G, Felicani C, Morselli-Labate AM: Clinical and patient-reported outcomes after pancreatoduodenectomy for different diseases: a follow-up study. Pancreas 2011, 40:938-945.

Rosso T, Malvezzi M, Bertuccio P, Negri E, La Vecchia, C, Decarli A: Cancer mortality in Italy, 2008, and predictions for 2012. Tumori 2012, 98:559-567.

Rozen P, Liphshitz I, Rosner G, Barchana M, Lachter J, Pel S, Shohat T, Santo E: Pancreatic cancer in Israel: the epidemiology, possibilities of prevention, early detection and screening. Isr Med Assoc J 2009, 11:710-713.

Rutegard M, Shore R, Lu Y, Lagergren P, Lindblad M: Sex differences in the incidence of gastrointestinal adenocarcinoma in Sweden 1970-2006. Eur J Cancer 2010, 46:1093-1100.

Sant M, Aareleid T, Berrino F, Bielska LM, Carli PM, Faivre J, Grosclaude P, Hedelin G, Matsuda T, Moller H, et al: EUROCARE-3: survival of cancer patients diagnosed 1990-94--results and commentary. Ann Oncol 2003, 14 Suppl 5:v61-118.

Soerjomataram I, de Vries, E, Pukkala E, Coebergh JW: Excess of cancers in Europe: a study of eleven major cancers amenable to lifestyle change. Int J Cancer 2007, 120:1336-1343.

Soreide K, Aagnes B, Moller B, Westgaard A, Bray F: Epidemiology of pancreatic cancer in Norway: trends in incidence, basis of diagnosis and survival 1965-2007. Scand J Gastroenterol 2010, 45:82-92.

Storm HH, Engholm G, Hakulinen T, Tryggvadottir L, Klint A, Gislum M, Kejs AM, Bray F: Survival of patients diagnosed with cancer in the Nordic countries up to 1999-2003 followed to the end of 2006. A critical overview of the results. Acta Oncol 2010, 49:532-544.

Talback M, Stenbeck M, Rosen M, Barlow L, Glimelius B: Cancer survival in Sweden 1960-1998--developments across four decades. Acta Oncol 2003, 42:637-659.

Teiblum S, Thygesen LC, Johansen C: Sixty-one years of pancreatic cancer in Denmark from 1943 to 2003: a nationwide study. Pancreas 2009, 38:374-378.

Tingstedt B, Andersson E, Flink A, Bolin K, Lindgren B, Andersson R: Pancreatic cancer, healthcare cost, and loss of productivity: a register-based approach. World J Surg 2011, 35:2298-2305.

Vaktskjold A, Lebedintseva JA, Korotov DS, Tkatsjov AV, Podjakova TS, Lund E: Cancer incidence in Arkhangelskaja Oblast in northwestern Russia. The Arkhangelsk Cancer Registry. BMC Cancer 2005, 5:82.

Vaktskjold A, Ungurjanu TN, Klestsjinov NM: Cancer incidence in the Nenetskij Avtonomnyj Okrug, Arctic Russia. Int J Circumpolar Health 2008, 67:433-444.

Valean S, Armean P, Resteman S, Nagy G, Muresan A, Mircea PA: Cancer mortality in Romania, 1955-2004. Digestive sites: esophagus, stomach, colon and rectum, pancreas, liver, gallbladder and biliary tree. J Gastrointestin Liver Dis 2008, 17:9-14.

Vercelli M, Capocaccia R, Quaglia A, Casella C, Puppo A, Coebergh JW: Relative survival in elderly European cancer patients: evidence for health care inequalities. The EUROCARE Working Group. Crit Rev Oncol Hematol 2000, 35:161-179.

Visser O, van Leeuwen FE: Stage-specific survival of epithelial cancers in North-Holland/Flevoland, The Netherlands. Eur J Cancer 2005, 41:2321-2330.

Wood HE, Gupta S, Kang JY, Quinn MJ, Maxwell JD, Mudan S, Majeed A: Pancreatic cancer in England and Wales 1975-2000: patterns and trends in incidence, survival and mortality. Aliment Pharmacol Ther 2006, 23:1205-1214.

Zabernigg A, Gamper EM, Giesinger JM, Rumpold G, Kemmler G, Gattringer K, Sperner-Unterweger B, Holzner B: Taste alterations in cancer patients receiving chemotherapy: a neglected side effect? Oncologist 2010, 15:913-920.

**Table S1: Characteristics of selected studies reporting epidemiological, clinical, and quality-of-life data**

| **Study** | **Study type** | **Country/Countries** | **Region classification** | **Name of cancer registry or registries (or main institution if non-registry)** | **Registry classification** | **Total study popn.** | **Study start** | **Study end** | **Study length (years)** |
| --- | --- | --- | --- | --- | --- | --- | --- | --- | --- |
| Aareleid 1991 | Database/registry analysis | Estonia | Single European country | Estonian Cancer Registry | Single national registry | NR | 1978 | 1987 | 10.0 |
| Adanja 1994 | Database/registry analysis | Yugoslavia (Belgrade) | Single European country | Unclear | Single national registry | NR | 1975 | 1989 | 15.0 |
| Allen-Mersh 1986 (database review) | Database/registry analysis | England, Wales | Single European country | Office of Population. Censuses and Surveys (OPCS), Hospital Activity Analysis (HAA) and Hospital In-Patient Enquiry (HIPE) | Multiple registries assessed | NR | 1979 |  |  |
| Appelqvist 1983 | Cohort study (retrospective/  prospective longitudinal) | Finland | Single European country | Department of Radiotherapy and Oncology, University Central Hospital, Helsinki, Finland | Non-registry (e.g. single institution) | 267 | 1947 | 1980 | 33.0 |
| Arfe 2011 | Database/registry analysis | Italy | Single European country | WHO mortality database | Single international registry (e.g. WHO) | NR | 1970 | 2007 | 37.0 |
| Arnar 1991 | Cohort study (retrospective/  prospective longitudinal) | Iceland | Single European country | Icelandic Cancer Registry, the disease registries of the four largest hospitals in Iceland, and the tumour registry of the Dept. of Pathology at the University of Iceland. | Single regional registry | 281 | 1974 | 1985 | 12.0 |
| Baastrup 2008 | Database/registry analysis | Denmark | Single European country | Various Danish registers, based on administrative data | Multiple registries assessed | NR | 1994 | 2003 | 10.0 |
| Bernal 2009 | Database/registry analysis | Spain | Single European country | Official Spanish database (unclear) | Single national registry | NR | 1975 | 2004 | 30.0 |
| Bjerregaard 2013 | Database/registry analysis | Southern Denmark | Single European country | Danish Cancer Registry | Single regional registry | 579 | 2007 | 2009 | 3.0 |
| Borras 2001 | Database/registry analysis | Spain (Tarragona) | Single European country | Tarragona Cancer Registry | Single regional registry | 8,558 | 1980 | 1996 | 17.0 |
| Bosetti 2012 | Database/registry analysis | 35 European countries and other 19 countries in the world | International (Europe & non-Europe) | World Health Organization and Pan American Health Organization (PAHO) | Single international registry (e.g. WHO) | NR | 1980 | 2007 | 17.0 |
| Bramhall 1995 | Database/registry analysis | UK (West Midlands) | Single European country | West Midlands Region Cancer Registry | Single regional registry | NR | 1957 | 1986 | 30.0 |
| Bray, 2000 | Database/registry analysis | Bulgaria, Czech Republic and Slovakia (analysed together), Hungary, Poland, and Romania | Multiple European countries | World Health Organization and Pan American Health Organization (PAHO) | Single international registry (e.g. WHO) | NR | 1965 | 1994 | 30.0 |
| Bye, 2013 | Cohort study (retrospective/  prospective longitudinal) | Norway | Single European country | Department of Oncology or the Unit for Palliative Care at Oslo University Hospital, Norway | Non-registry (e.g. single institution) | 39 | Aug  2006 | Aug 2008 | 2.0 |
| Carpelan-Holmstrom 2005 | Database/registry analysis | Finland | Single European country | Finnish Cancer Registry and Statistics Finland | Single national registry | 4,922 | 1990 | 1996 | 7.0 |
| Coupland 2012 | Database/registry analysis | England | Single European country | National Cancer Data Repository | Single regional registry | 99,397 | 1998 | 2007 | 10.0 |
| Crocetti 2004 | Database/registry analysis | Italy | Single European country | Network of the Italian Cancer Registries (AIRT) and National Institute of Statistics (ISTAT) for mortality rates | Single national registry | NR | 1986 | 1997 | 12.0 |
| Cronin-Fenton 2011 | Cohort study (retrospective/  prospective longitudinal) | Central and Northern Denmark | Single European country | Danish National Patient Registry (DNPR) | Single national registry | 2,968 | 1998 | 2009 | 12.0 |
| Cylwik 1984 | Cohort study (retrospective/  prospective longitudinal) | Bialystok, Poland | Single European country | All hospitals of Biarystok (the administrative, industrial and cultural centre of north-eastern region of Poland) | Multiple registries assessed | 18,751 | 1953 | 1982 | 30.0 |
| Decarli 1987 | Database/registry analysis | Italy | Single European country | Department of Demography of the University of Rome (1955-1972), and by the Central Institute of Statistics (ISTAT) from 1972 onward | Single national registry | NR | 1955 | 1979 | 25.0 |
| Desauw 2012 | Database/registry analysis | France (Lille) | Single European country | Cancer registry of Lille | Single regional registry | 260 | 2005 or 2008 | 2005 or 2008 | 1.0 |
| Downing 2008 | Database/registry analysis | UK (Yorkshire) | Single European country | Northern & Yorkshire Cancer Registry | Single regional registry | NR | 1983 | 2003 | 20.0 |
| Faivre 1998 | Database/registry analysis | 17 European countries (Iceland, Finland, Sweden, Denmark, Scotland, England, the Netherlands, Germany, Austria, Switzerland, France, Spain, Italy, Slovenia, Slovakia, Poland, Estonia) | Multiple European countries | Registries of Finland, Denmark, Iceland, Estonia, Slovenia and Slovakia cover the entire population of these countries. U.K. registries cover approximately 50% of the whole population. Other countries are represented by one or more regional registries | Multiple registries assessed | 53,898 | 1978 | 1989 | 12.0 |
| Fernandez 1994 | Database/registry analysis | EU (25) Austria, Belgium, Bulgaria, Czechoslovakia, Denmark, Finland, France, Germany GDR, Germany FRG, Greece, Hungary, Iceland, Ireland, Italy, Netherlands, Norway, Poland, Portugal, Spain, Sweden, Switzerland, UK, England and Wales, UK, UK, Northern Ireland, UK, Scotland, Yugoslavia | Multiple European countries | WHO | Single international registry (e.g. WHO) | NR | 1955 | 1989 | 35.0 |
| Fitzsimmons 2006 | Cohort study (retrospective/  prospective longitudinal) | England, Wales | Single European country | Office of National Statistics for stomach and pancreatic cancer | Single national registry | 21 | 1951 | 2000 | 50.0 |
| Fitzsimmons 1999 | Cohort study (retrospective/  prospective longitudinal) | UK (Wessex Region) | Single European country | Interviews | Non-registry (e.g. single institution) | NR | Dec  1995 | Sept 1996 | 10 months |
| Frigeri 2012 | Cohort study (retrospective/  prospective longitudinal) | Switzerland | Single European country | Oncology Institute of Southern Switzerland | Single regional registry | NR | 1993 | 2010 | 18.0 |
| Gencer 2009 | Cohort study (retrospective/  prospective longitudinal) | Germany | Single European country | University of Heidelberg | Non-registry (e.g. single institution) | 435 | Jun  1998 | Dec 2006 | 8.5 |
| Grieve 1973 | Cohort study (retrospective/  prospective longitudinal) | UK (Edinburgh) | Single European country | Western general hospital | Non-registry (e.g. single institution) | 100 | 1956 | 1970 | 15.0 |
| Hakama 1975 | Database/registry analysis | Finland | Single European country | Finnish Cancer Registry | Single national registry | NR | 1953 | 1970 | 18.0 |
| Hariharan 2008 | Database/registry analysis | Europe (33), Asia (10), America (8). 51 countries (Belgium, France, Germany, Ireland, Luxemburg, The Netherlands, Switzerland and the United Kingdom, Bulgaria, Estonia, Latvia, Lithuania, Moldova, Poland, Romania, Denmark, Finland, Iceland, Norway and Sweden, Albania, Croatia, Greece, Italy, Macedonia, Malta, Portugal, Slovenia and Spain, Austria, Czechoslovakia, Hungary Slovakia, Hong Kong, Philippines Singapore, Japan Korea, Georgia, Kyrgyzstan, Russia, Israel, Kuwait, Canada, USA, Argentina, Chile, Costa Rica, Mexico, Uruguay, Venezuela ) | International (Europe & non-Europe) | Database from International Agency for Research on Cancer- CANCER Mondial Statistical Information System | Single international registry (e.g. WHO) |  | 1992 | 2002 | 10.0 |
| Haynes 2009 | Database/registry analysis | UK | Single European country | UK cancer registry for England and Wales/ The Health Improvement Network (THIN) database | Single regional registry |  | 1992 | 2007 | 15.0 |
| Hedberg 1998 | Database/registry analysis | Southern Sweden (Lund) | Single European country | Local Tumour Register in Lund, Sweden | Single regional registry |  | 1977 | 1991 | 15.0 |
| Hill 1991 | Database/registry analysis | France | Single European country | Unclear | Multiple registries assessed |  | 1950 | 1985 | 35.0 |
| Hippisley-Cox 2012 | Cohort study (retrospective/  prospective longitudinal) | England and Wales | Single European country | QResearch database (version 30) | Single regional registry | 4,149,461 | 1 Jan 2000 | 30 Sept 2010 | 10 years and 10 months |
| Hiripi, 2012 | Database/registry analysis | Germany | Single European country | 11 population-based German cancer registries, Saarland Cancer registry | Multiple registries assessed |  | 1997 | 2006 | 10.0 |
| Hjelmgren 2003 | Cohort study (retrospective/  prospective longitudinal) | Sweden | Single European country | Patient records from 4 hospitals were chosen: the university hospitals (UH) in Lund and Malmo and the regional/local hospitals in Helsingborg and Kristianstad, Swedish Cancer Registry | Multiple registries assessed | 53 | 1997 | 1999 | 2.0 |
| Jacobsen 1985 | Cohort study (retrospective/  prospective longitudinal) | Faroe Islands | Single European country | Records the three hospitals of Faroe Islands | Single regional registry | 57 | 1972 | 1982 | 10.0 |
| Jooste 2011 | Database/registry analysis | France | Single European country | French population-based cancer registries, biostatistics department of the Hospices Civils de Lyon, the InVS, and the National Cancer Institute | Multiple registries assessed |  | 1980 | 2005 | 25.0 |
| Karim-Kos 2008 | Database/registry analysis | 21 European countries (Denmark, Finland, Norway, Sweden, Ireland, the United Kingdom, Austria, France, Germany, The Netherlands, Switzerland, Croatia, Italy, Malta, Slovenia, Spain, Czech Republic, Lithuania, Poland) | Multiple European countries | National or regional cancer registry websites or annual reports. | Multiple registries assessed |  | mid 1990 | mid 2000 | 10.0 |
| Karim-Kos 2011 | Database/registry analysis | The Netherlands | Single European country | Netherlands Cancer Registry (NCR, Statistics Netherlands | Multiple registries assessed |  | 1989 | 2009 | 20.0 |
| Kirkegaard 2012 | Database/registry analysis | Greenland and Denmark | Multiple European countries | Danish Cancer Register and The Greenlandic Patient Register | Single national registry |  | 2000 | 2010 | 10.0 |
| Klint 2010 | Database/registry analysis | Nordic countries: Denmark Finland Iceland Norway Sweden | Multiple European countries | NORDCAN database + national cancer registries | Multiple registries assessed |  | 1964 | 2003 | 40.0 |
| La Rosa 1997 | Cross-sectional | Italy (Umbria) | Single European country | Case records of all diagnostic and therapeutic services public and private hospitals in the region and those of main national oncological centres + regional registry of mortality RENCAM , regional list of people covered by the National Health Service | Multiple registries assessed |  | 1978 | 1982 | 4.0 |
| Labori 2006 | Cohort study (retrospective/  prospective longitudinal) | Norway | Single European country | Ullevaal University Hospital, Norway | Non-registry (e.g. single institution) | 51 | May 2003 | June 2005 | 2.0 |
| Lambe 2011 | Database/registry analysis | Sweden | Single European country | Swedish Cancer Register, the National Patient Register, and the Cause of Death Register, at the Centre of Epidemiology at the Swedish National Board of Health and Welfare. | Multiple registries assessed | 4321 | 1987 | 1999 | 12.0 |
| Lepage 2006 | Cohort study (retrospective/  prospective longitudinal) | France | Single European country | National Institute for Health and Medical Research (INSERM). | Single national registry |  | 1980 | 2000 | 20.0 |
| Levi 1997 | Database/registry analysis | Switzerland | Single European country | Swiss Federal Statistical Office | Single national registry |  | 1990 | 1994 | 5.0 |
| Levi 1987 | Database/registry analysis | Switzerland | Single European country | (Swiss Federal Office of Statistics-SFOS- | Single national registry |  | 1951 | 1984 | 33.0 |
| Levi 2001 | Database/registry analysis | 23 European countries/ US/ Japan | International (Europe & non-Europe) | World Health Organization (WHO) database | Single international registry (e.g. WHO) | 31158 | 1960 | 1998 | 38.0 |
| Levi 2003 | Database/registry analysis | (16 EE countries and 6 eastern Europe countries): 22 countries (Austria, Belgium, Denmark, Finland, France, Germany, Greece, Ireland, Italy, Luxembourg, Netherlands, Norway, Portugal, Spain, Sweden, UK, Bulgaria, Czech Republic, Hungary, Poland, Romania, Slovakia) | Multiple European countries | WHO database (http://www3.who.int/whosis/menu.cfm) | Single international registry (e.g. WHO) |  | 1980 | 1999 | 19.0 |
| Levi 2000 | Database/registry analysis | Switzerland (Vaud) | Single European country | Vaud Cancer Registry | Single regional registry |  | 1974 | 1993 | 19.0 |
| Levi 2003 | Database/registry analysis | EU (15) Austria, Belgium, Denmark, Finland, France, Germany, Greece, Ireland, Italy, Luxembourg, The Netherlands, Portugal, Spain, Sweden, UK | Multiple European countries | WHO | Single international registry (e.g. WHO) |  | 1955 | 1998 | 43.0 |
| Linder 2007 | Database/registry analysis | Sweden | Single European country | Swedish Hospital Discharge Register and Cancer Register and Register of Causes of Death in Sweden | Multiple registries assessed | 16758 | 1980 | 2000 | 20.0 |
| Liszka 2010 | Cross-sectional (e.g. survey) | Poland | Single European country | Medical University of Silesia, Katowice | Non-registry (e.g. single institution) | 580 | 1985 | 2009 | 25.0 |
| Lombard-Bohas 2008 | Cross-sectional (e.g. survey) | France | Single European country | Fédération Francophone de Cancérologie Digestive (FFCD), Association des hépato-gastroentérologues des hôpitaux généraux (ANGH) and Groupe cooperateur multidisciplinaire en oncology (GERCOR)]. | Non-registry (e.g. single institution) | 668 | Aug  2001 | Sept 2002 | 13 months |
| Luo 2007 | Database/registry analysis | Sweden | Single European country | Swedish Cancer Register | Single national registry |  | 1960 | 2003 | 43.0 |
| Markovic-Denić 2008 | Database/registry analysis | Central Serbia | Single European country | Death certificates (Federal Institute of Statistics) | Single national registry |  | 1985 | 2006 | 21.0 |
| Malvezzi 2011 | Database/registry analysis | EU (27 countries) Austria, Belgium, Bulgaria, the Czech Republic, Cyprus, Denmark, Estonia, Finland, France, Germany, Greece, Hungary, Ireland, Italy, Latvia, Lithuania, Luxembourg, Malta, the Netherlands, Poland, Portugal, Romania, Slovakia, Slovenia, Spain, Sweden, UK | Multiple European countries | World Health Organization and EUROSTAT | Single international registry (e.g. WHO) |  | 1970 | 2008 | 38.0 |
| Malvezzi 2012 | Database/registry analysis | EU (27 countries) Austria, Belgium, Bulgaria, the Czech Republic, Cyprus, Denmark, Estonia, Finland, France, Germany, Greece, Hungary, Ireland, Italy, Latvia, Lithuania, Luxembourg, Malta, the Netherlands, Poland, Portugal, Romania, Slovakia, Slovenia, Spain, Sweden, UK | Multiple European countries | World Health Organization and EUROSTAT | Single international registry (e.g. WHO) |  | 1970 | 2009 | 39.0 |
| Matos 2009 | Cohort study (retrospective/prospective longitudinal) | Germany, USA | Multiple countries | Indiana University School of Medicine Institutional Review Board (IRB) respective institution’s (Dresden, Mannheim, and Vanderbilt) IRB | Non-registry (e.g. single institution) |  | 1988 | 2008 | 20.0 |
| Micheli 2009 | Database/registry analysis | 23 European countries | Multiple European countries | EUROCARE-4 dataset which contains standardised population-based information on about 3 million cancer cases from 82 cancer registries (CRs) in 23 European countries | Single international registry (e.g. WHO) | 1,668,872 | 1995 | 1999 | 5.0 |
| Mitry 2008 | Database/registry analysis | England, Wales | Single European country | Unclear | Single national registry |  | 1986 | 1999 | 13.0 |
| Moulard 2012 | Database/registry analysis | France, Italy, Germany, Netherlands, Austria, Norway, Ireland, Finland, UK | Multiple European countries | Nine registries (in France, Italy, Germany, Netherlands, Austria, Norway, Ireland, Finland, UK) | Multiple registries assessed |  | 2000 | 2012 | 13.0 |
| Mukherjee 2008 | Cohort study (retrospective/  prospective longitudinal) | Wales | Single European country | Velindre Cancer Centre + (Information System for Clinical Organisations, ISCO | Multiple registries assessed | 315 | 2002 | 2005 | 4.0 |
| Müller-Nordhorn 2006 | Cohort study (retrospective/  prospective longitudinal) | Berlin, Germany | Single European country | Charité University Medical Center, | Non-registry (e.g. single institution) | 57 | Dec 2000 | Feb  2002 | 2.0 |
| Nagenthiraja 2007 | Database/registry analysis | Nordic Countries: Denmark, Finland, Iceland, Norway, Sweden | Multiple European countries | Cancer registries in Denmark, Finland, Iceland, Norway, Sweden / NORDCAN program | Multiple registries assessed |  | 1971 | 2000 | 30.0 |
| Nienhuijs 2012 | Cohort study (retrospective/  prospective longitudinal) | The Netherlands | Single European country | Netherlands Cancer Registry trends (NCR) | Single national registry |  | 1989 | 2008 | 20.0 |
| Pasquali 2002 | Cohort study (retrospective/  prospective longitudinal) | Northern Italy (Veneto Region) | Single European country | Unclear | Multiple registries assessed |  | 1990 | 1992 | 3.0 |
| Patterson 1991 | Database/registry analysis | Northern Ireland | Single European country | Registrar General's Office | Single regional registry |  | 1979 | 1988 | 10.0 |
| Pezzilli 2011 | Cohort study (retrospective/  prospective longitudinal) | Italy (Verona, Bologna, Milan) | Single European country | Sant’Orsola-Malpighi Hospital of Bologna, G. B. Rossi Hospital of Verona, San Raffaele Hospital of Milan | Multiple registries assessed | 197 | Feb  2006 | Mar 2007 | 2.0 |
| Rosso 2012 | Database/registry analysis | Italy | Single European country | World Health Organization (WHO) database (WHOSIS) | Single international registry (e.g. WHO) |  | 2008 | 2008 | 1.0 |
| Rozen 2009 | Database/registry analysis | Israel | Single non European country | Israel National Cancer Registry | Single national registry |  | 1980 | 2006 | 26.0 |
| Rutegard 2010 | Database/registry analysis | Sweden | Single European country | Swedish Cancer Register | Single national registry |  | 1970 | 2006 | 36.0 |
| Sant 2003 | Database/registry analysis | 22 European countries (Austria, Czech Republic, Denmark, England, Estonia, Finland, France, Germany, Iceland, Italy, Malta, Netherlands, Norway, Poland, Scotland, Slovakia, Slovenia, Spain, Sweden, Switzerland, Wales) | Multiple European countries | 67 population-based cancer registries | Multiple registries assessed |  | 1990 | 1994 | 5.0 |
| Soerjomataram 2007 | Database/registry analysis | 28 European countries (Austria, Belgium, Bulgaria, Czech Republic, Denmark, Estonia, Finland, France, Germany, Greece, Hungary, Ireland, Italy, Latvia, Lithuania, Former Yugoslavian Republic of Macedonia, the Netherlands, Norway, Poland, Portugal, Romania, Serbia and Montenegro, Slovakia, Slovenia, Spain, Sweden, Switzerland and UK) | Multiple European countries | Eurostat and GLOBOCAN 2002 | Multiple registries assessed |  | 2002 | 2002 | 1.0 |
| Soreide 2010 | Database/registry analysis | Norway | Single European country | Cancer Registry of Norway | Single national registry |  | 1965 | 2007 | 43.0 |
| Storm 2010 | Database/registry analysis | Nordic countries (Denmark Finland Iceland Norway Sweden) | Multiple European countries | NORDCAN database | Single international registry (e.g. WHO) |  | 1999 | 2003 | 5.0 |
| Talback 2003 | Database/registry analysis | Sweden | Single European country | Swedish Cancer Registry | Single national registry | 1,021,421 | 1960 | 1998 | 38.0 |
| Teiblum 2009 | Database/registry analysis | Denmark | Single European country | Danish Cancer Registry | Single national registry |  | 1943 | 2003 | 61.0 |
| Tingstedt, 2011 | Cohort study (retrospective/prospective longitudinal) | Sweden | Single European country | Lund University Hospital, Sweden | Non-registry (e.g. single institution) | 83 | 2005 | 2007 | 3.0 |
| Vaktskjold 2005 | Database/registry analysis | Arkhangelskaja, Northwestern Russia | Single European country | Arkhangelskaja Cancer registry | Single regional registry |  | 1993 | 2001 | 8.0 |
| Vaktskjold 2008 | Database/registry analysis | Arkhangelskaja Oblast/ north-west Russia | Single European country | Central Oncology Hospital (AOKOD) of Arkhangelskajan Oblast | Single regional registry |  | 1993 | 2006 | 14.0 |
| Vălean 2008 | Database/registry analysis | Romania | Single European country | Statistics of IARC/OMS, Lyon, France and from the Ministry of Public Health, Bucharest, Romania | Multiple registries assessed |  | 1955 | 2004 | 50.0 |
| Vercelli 2000 | Database/registry analysis | 16 European countries (Iceland, Finland, Sweden, Denmark, UK, The Netherlands, Germany, Austria, Switzerland, France, Italy, Spain, Slovenia, Slovakia, Poland, Estonia) | Multiple European countries | 44 population-based cancer registries in 16 European countries | Multiple registries assessed |  | 1985 | 1989 | 5.0 |
| Visser 2005 | Database/registry analysis | The Netherlands (North-Holland/Flevoland) | Single European country | Amsterdam Cancer Registry | Single regional registry |  | 1989 | 2001 | 12.0 |
| Wood 2006 | Database/registry analysis | England, Wales | Single European country | 9 regional registries in England and the Welsh Cancer Intelligence and Surveillance Unit in Wales / Local Registration Service in partnership with the General Register Office (death registry) | Single regional registry |  | 1975 | 2000 | 25.0 |
| Zabernigg 2010 | Cohort study (retrospective/  prospective longitudinal) | Austria | Single European country | Department of Internal Medicine at Kufstein County Hospital (Teaching Hospital of Innsbruck Medical University) | Non-registry (e.g. single institution) | 197 | Apr  2007 | June 2009 | 2.3 |

NR: Not reported

**Figure S1. Survival at 1 and 5 years by country (sexes combined)**


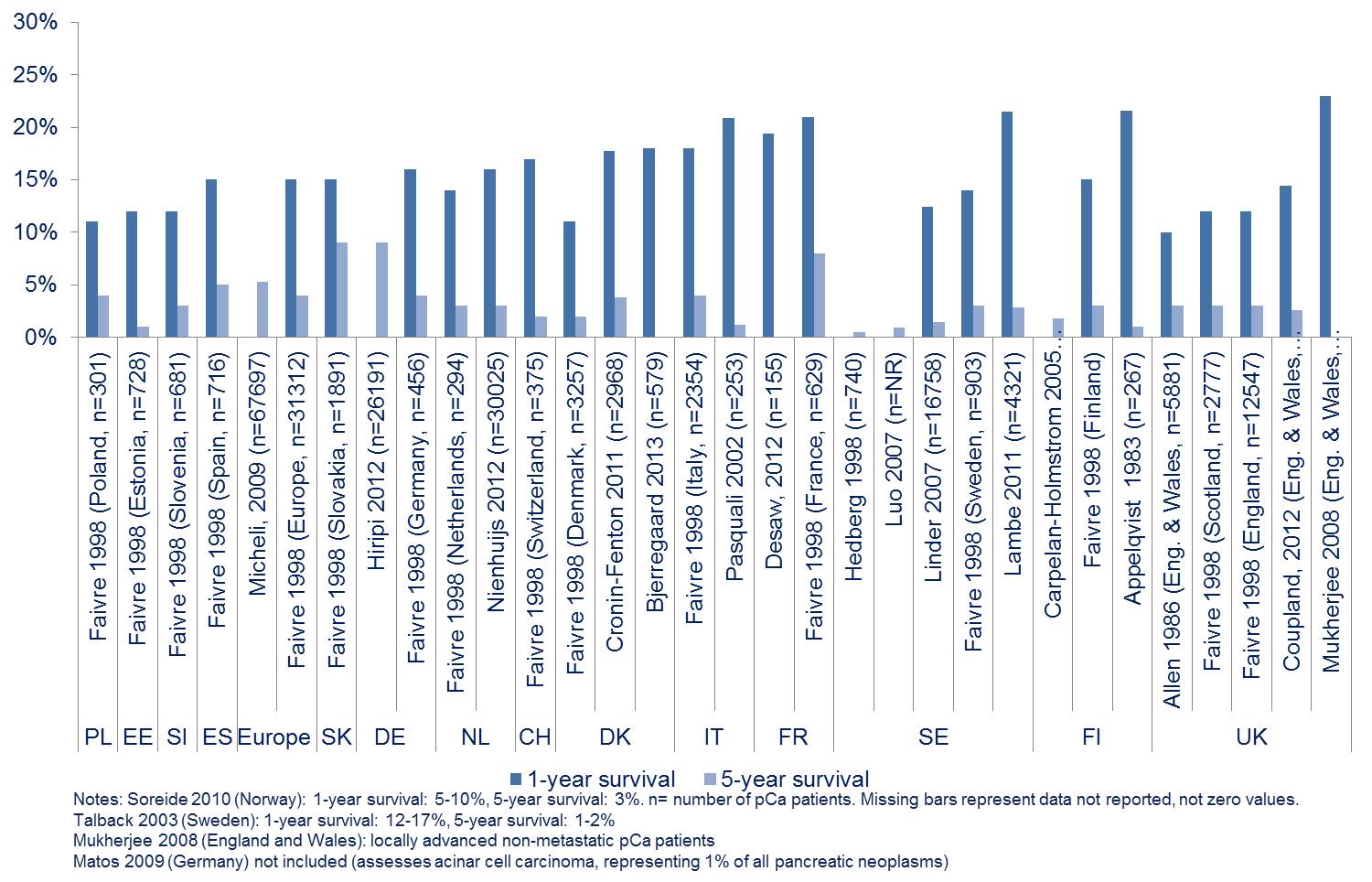


**Figure S2. Median survival from diagnosis by intervention received**

**
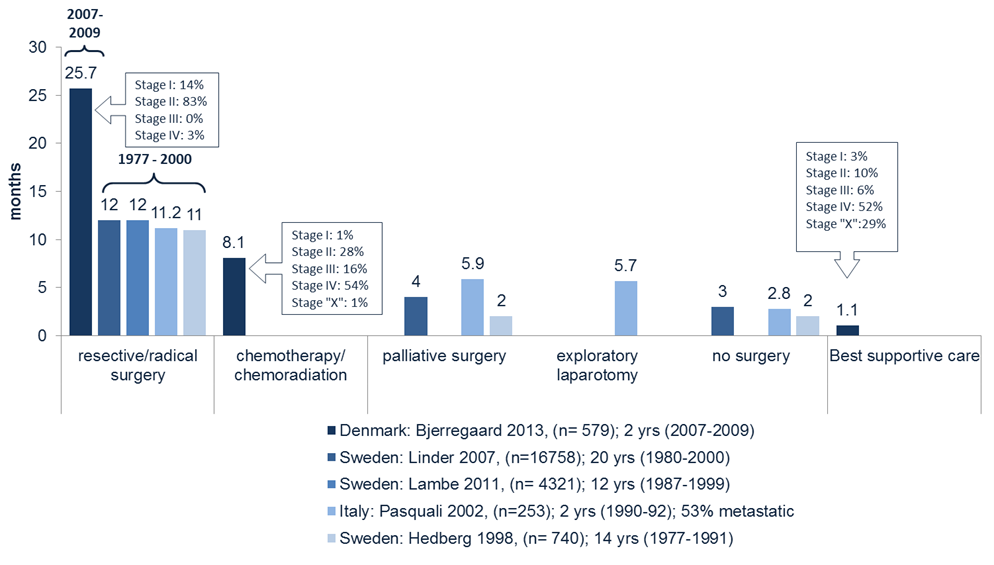
**
